# Construction of dynamic probabilistic protein interaction networks for protein complex identification

**DOI:** 10.1186/s12859-016-1054-1

**Published:** 2016-04-27

**Authors:** Yijia Zhang, Hongfei Lin, Zhihao Yang, Jian Wang

**Affiliations:** College of Computer Science and Technology, Dalian University of Technology, Dalian, Liaoning 116023 China

**Keywords:** Dynamic networks, Gene expression data, Protein complex identification, Protein-protein interaction networks

## Abstract

**Background:**

Recently, high-throughput experimental techniques have generated a large amount of protein-protein interaction (PPI) data which can construct large complex PPI networks for numerous organisms. System biology attempts to understand cellular organization and function by analyzing these PPI networks. However, most studies still focus on static PPI networks which neglect the dynamic information of PPI.

**Results:**

The gene expression data under different time points and conditions can reveal the dynamic information of proteins. In this study, we used an active probability-based method to distinguish the active level of proteins at different active time points. We constructed dynamic probabilistic protein networks (DPPN) to integrate dynamic information of protein into static PPI networks. Based on DPPN, we subsequently proposed a novel method to identify protein complexes, which could effectively exploit topological structure as well as dynamic information of DPPN. We used three different yeast PPI datasets and gene expression data to construct three DPPNs. When applied to three DPPNs, many well-characterized protein complexes were accurately identified by this method.

**Conclusion:**

The shift from static PPI networks to dynamic PPI networks is essential to accurately identify protein complex. This method not only can be applied to identify protein complex, but also establish a framework to integrate dynamic information into static networks for other applications, such as pathway analysis.

## Background

Recent advances in high-throughput experimental techniques such as yeast two-hybrid and mass spectrometry have generated a large amount of protein-protein interaction (PPI) data [1, 2]. These available PPI data have constructed large complex PPI networks for numerous organisms, such as *Saccharomyces cerevisiae*. PPIs are of central importance for most biological processes, and thus PPI networks can provides a global picture of cellular mechanisms. A key task of system biology is to reveal cellular organization and function by analyzing the PPI networks. Protein complexes are molecular aggregations of two or more proteins assembled by multiple PPIs, which play critical roles in many biological processes. Most proteins are only functional after assembly into protein complexes. Accurate determination of protein complexes in large PPI networks is crucial for understanding principles of cellular organization and function from the networks level [3].

Over the past decade, great effort has been made to identify protein complexes in PPI networks. As protein complexes are groups of proteins that interact with each other, they are generally dense subgraph in PPI networks. Some computational methods based on graph theory or dense regions finding have been proposed to identify protein complexes from PPI networks. The molecular complex detection (MCODE [4]) algorithm proposed by Bader and Hogue was one of the first computational methods reported based on graph theory. Markov Clustering (MCL) [5] can also be applied to identify protein complexes by simulating random walks in PPI networks, which manipulates the weighted or unweighted adjacency matrix with two operators called expansion and inflation. Qi et al*.* [6] proposed a supervised-learning framework to predict protein complexes, which can learn topological and biological features from known protein complexes. Adamcsek et al*.* [7] developed the CFinder tool to find functional modules in PPI networks, which use the clique percolation method [8] to detect k-clique percolation clusters. Moschopoulos et al*.* proposed a clustering tool (GIBA) to detect protein complexes [9], which involves two phases. Firstly, GIBA uses a clustering algorithm such as MCL and RNSC to cluster the given PPI networks. Then, GIBA filters the clustering results to generate the final complexes based on a combination method. Liu et al*.* [10] proposed a clustering method based on Maximal cliques (CMC) to detect protein complexes. Based on core-attachment structural features [11], Wu et al*.* [12] developed the COACH algorithm which identifies protein-complex cores and protein-complex attachments respectively. Zaki et al*.* proposed ProRank method which uses a protein ranking algorithm to identify essential proteins in a PPI network and predicts complexes based on the essential proteins [13]. Chin et al*.* proposed a hub-attachment based method called HUNTER to detect functional modules and protein complexes from confidence-scored protein interactions [14]. Since proteins may have multiple functions, they may belong to more than one protein complex. Nepusz et al*.* [15] proposed the ClusterONE algorithm which detected overlapping protein complexes in PPI networks. High-throughput experimental PPI data always is the high incidence of both false positives and false negatives [3]. Since the computational methods are highly dependent on the quality of the PPI data, the performance of complex predictive models are clearly limited by the noise of the high-throughput PPI data. Some studies have integrated other biomedical resources to improve the performance of protein complex identification. For instance, Zhang et al*.* [16] proposed the COAN algorithm based on ontology augmentation networks constructed with high-throughput PPI and gene ontology (GO) annotation data, which can takes into account the topological structure of the PPI network, as well as similarities in GO annotations.

So far most studies on protein complex identification only focused on static PPI networks. However, cellular systems are highly dynamic and responsive to cues from the environment [17, 18]. PPI network in a cell changes over time, environments and different stages of cell cycle [19, 20]. PPIs can be classified into permanent or transient PPIs based on their lifetime. Permanent PPIs are usually stable and irreversible. On the contrary, transient PPIs mostly dynamical change interaction partners and their lifetime are short. Protein complexes are groups of two or more associated polypeptide chains at the same time. One major problem of protein complex identification is the static PPI networks cannot provide temporal information and do not reflect the actual situation in a cell [21]. It is very difficult to identify complex accurately from the static PPI networks.

To address this problem, the shift from static PPI networks to dynamic PPI networks is essential for protein complex identification and other similar applications. The gene expression data under different time points and conditions can reveal the dynamic information of protein. Some studies have integrated gene expression data to reveal the dynamics of PPI. For example, Lin et al*.* [22] revealed dynamic functional modules under conditions of dilated cardiomyopathy based on co-expression PPI networks. Taylor et al*.* [23] analyzed the human PPI networks and discovered two types of hub proteins: intermodular hubs and intramodular hubs. Zhang et al*.* [24] used the Pearson correlation coefficient to calculate the coexpression correlation of gene expression data and built coexpression protein networks at different time points. Recently, Hanna et al*.* proposed a framework termed DyCluster to detect complexes based on PPI networks and gene expression data [25]. Firstly, DyCluster uses biclustering techniques to model the dynamic aspect of PPI networks by incorporating gene expression data. Then, DyCluster applies complex-detection algorithms, such as ClusterONE [15] and CMC [10], to detect the complexes from the dynamic PPI networks.

In general, the inevitable background noise exists in the gene expression data. How to identify the active time point of each protein based on gene expression data is crucial for constructing dynamic PPI networks. In this study, we proposed a novel method to calculate the active probability of proteins at different time points. Furthermore, we constructed dynamic probabilistic PPI networks (DPPN) to integrate gene expression data and PPI data based on attributed graph theory, and proposed a clustering method to identify protein complex from DPPN. There are two key differences between our method and DyCluster. Firstly, the DPPN constructed by our method can effectively distinguish the active level of a protein at a time point which is of benefit to the complex identification. Secondly, our method doesn’t directly apply other complex-detection algorithms, but proposes a new clustering method for the characteristics of DPPN. We demonstrated the utility of the method by applying it to three different yeast PPI datasets and gene expression data. Three DPPNs were constructed and many well-characterized protein complexes were accurately identified. In addition, the method was compared with current protein complexes identification methods. The advantages of the method, potential applications and improvements were discussed.

## Methods

### Calculation of active probability for proteins

Since a protein has its active periods in the cell [17, 18], the protein and its interactions appear and disappear in the PPI networks in a living cell. Gene expression data can reflect the dynamic information of proteins varying with the time points or conditions. In general, the expression level of a protein will be decreased after the protein has completed its function. Therefore, a protein is active at the time point, when the related gene expression data is at the high level.

A simple idea is to use a single global threshold for identifying the active time point of each protein. If the gene expression value of a gene is higher than the global threshold at a time point, the gene is considered as expressed at that time point. However, the expression level of genes in activity period is different. Wang et al*.* [26] proposed a three-sigma method to identify active time points of each protein in a cellular cycle. The standard deviation (SD) is a statistical value which can measure how data are dispersed around their average. Let *X* be a real random variable of normal distribution *N*(*α*,σ^2^), which describes for each individual gene its distribution of gene expression values across time. For any *k* > 0, P{|*X* − *α*| < *kσ*} = 2Φ(*k*) − 1, where Φ(.) is the distribution function of the standard normal law. In particular, for k = 1, 2, 3 it follows that P{|*X* ‐ *α*| < *σ*} = P{*α* − *σ* < *X* < *α* + *σ*} ≈ 0.6827, P{|*X* ‐ *α*| < 2*σ*} ≈ 0.9545 and P{|*X* ‐ *α*| < 3*σ*} ≈ 0.9973. Based on the above empirical rules, Wang et al*.* [26] designed an active threshold for each gene by calculating its own characteristic gene expression data, and constructed dynamic PPI networks. Then, they tested some complex prediction methods, such as MCL [5], on the dynamic PPI networks. In this paper, we proposed a novel method to construct DPPN based on the three-sigma method [26]. Compared with the three-sigma method [26], our method can effective distinguish the active level of a protein at a time point. Furthermore, we also proposed a new clustering method to identify complexes for the characteristics of DPPN.

In fact, gene expression data always includes inevitable noise. The active proteins with low expression values are likely to be filtered out even though using an active threshold for each gene. To deal with this problem, we calculate the active probability of each protein at different time points based on three-sigma method. Gene expression data often contain expression profiles of *n* time points. Let G_i_(*p*) be the gene expression value of gene *p* at the time point *i*. Let *α*(*p*) and σ(*p*) be the algorithmic mean and SD of gene expression data G(*p*), respectively.

1 $$ \alpha (p)=\frac{{\displaystyle {\sum}_{i=1}^n{G}_i(p)}}{n} $$

2 $$ \sigma (p)=\sqrt{\frac{{\displaystyle {\sum}_{i=1}^n{\left({G}_i(p)\hbox{-} \alpha (p)\right)}^2}}{n\hbox{-} 1}} $$

Since different genes correspond to different expression curves, we calculate the active probability of a protein based on the algorithmic mean and SD of the corresponding gene. Firstly, the *k*-sigma (*k* = 1,2,3) threshold can be calculated based three-sigma method [20] as follows:

3 $$ Ge\_ thres{h}_k(p)=\alpha (p)+k\cdotp \sigma (p)\cdotp \left(1-\frac{1}{1+{\sigma}^2(p)}\right) $$

*Ge_thres*_*k*_ is the active threshold of gene *p* which is determined by the values of *α*(*p*),σ^2^(*p*) and *k* (the times of sigma). If σ^2^(*p*) is very low, it indicates that the fluctuation of the expression curve of gene *p* is also very small and the value of G_i_(*p*) tends to be very close to *α*(*p*). In this case, the value of *Ge_thresh*_*k*_ is close to *α*(*p*). If σ^2^(*p*) is very high, it indicates that the value of G_i_(*p*) is spread out over a large range of values. A large σ^2^(*p*) generally indicates much noise in the gene expression data of gene *p*. In this case, the value of *Ge_thresh*_*k*_ is close to *α*(*p*) + *k* · *σ*(*p*). Note that the range of *k* (the times of sigma) is in (0, 3), while 3 is the maximum times of sigma. The larger *k* is, the higher *Ge_thresh*_*k*_ gets. If we choose a larger *k*, the active proteins filtered by *Ge_thresh*_*k*_ will be with higher confidence. For instance, based on three-sigma rules, when G_i_(*p*)> *α*(*p*) + 3 · *σ*(*p*), the probability that the protein *p* (product of gene *p*) is active at the *i* time point is 99.7 %, but when G_i_(*p*) > *α*(*p*) + σ(*p*), the probability that the protein *p* (product of gene *p*) is active at the *i* time point is only 68.3 %. Based on the *Ge_thresh*_*k*_, we calculate the active probability of a protein in the *i* time point as follows.

4 $$ { \Pr}_i(p)=\left\{\begin{array}{cc}\hfill 0.99\hfill & \hfill if\ {G}_i(p)\ge Ge\_ thres{h}_3(p)\hfill \\ {}\hfill \begin{array}{c}\hfill 0.95\hfill \\ {}\hfill 0.68\hfill \\ {}\hfill 0\hfill \end{array}\hfill & \hfill \begin{array}{c}\hfill if\ Ge\_ thres{h}_3(p)>{G}_i(p)\ge Ge\_ thres{h}_2(p)\hfill \\ {}\hfill if\ Ge\_ thres{h}_2(p)>{G}_i(p)\ge Ge\_ thres{h}_1(p)\hfill \\ {}\hfill if\ {G}_i(p)<Ge\_ thres{h}_1(p)\hfill \end{array}\hfill \end{array}\right. $$

In the equation (4), the active probability of a protein contains four levels based on the sigma rules (P{|*X* ‐ *α*| < *σ*} ≈ 0.6827, P{|*X* ‐ *α*| < 2*σ*} ≈ 0.9545 and P{|*X* ‐ *α*| < 3*σ*} ≈ 0.9973). In particular, if the value of G_i_(*p*) is lower than *Ge_thres*_*1*_(*p*), the active probability is 0. This indicates that the protein *p* is not active in the *i* time point. In general, the active probability value of a protein can represent its active level at a time point. Thus, we can distinguish the active level of a protein at a time point based on its active probability. Neither global threshold method nor active threshold method can effectively distinguish the active level of a protein at a time point based on gene expression data. Based on the active probability of a protein, we can not only effectively identify the active time point of the protein, but also distinguish the active level of the protein.

### Construction of DPPN

Since the active periods of proteins are different, the real PPI networks are changing over the time in a living cell. We can calculate the active probability of proteins at each time point based on gene expression data. In this section, we construct DPPN by integrating the active information of proteins into static PPI networks based on attributed graph theory.

We define a DPPN as a 7-tuple *G* = (*V*, *E*, *A*, *P*, *Fv*, *Fe*, *Fp*) where *V* is the set of protein vertices, _*E*_ is the set of PPIs, *A* = {*T*1, *T*2, … *Tn*} is the set of active time points for protein vertices, and *P* = {*P*1, *P*2, *P*3} is the set of active probability for protein vertices at each active time point. *F*_v_ is a function that returns the set of active time attributes of a protein vertex. Each protein vertex *v*_*i*_ in *V* has a set of active time attributes *F*_*v*_(*v*_*i*_) = {*T*_*i*__1_, *T*_*i*__2_, …, *T*_*im*_}, where *m* = |*F*_*v*_(*v*_*i*_)| and *F*_*v*_(*v*_*i*_) ⊆ *A*. Likewise, *Fp*(*v*_*i*_, *T*_*ij*_) = *Pk* is a function that returns active probability *P*_*k*_ for the protein vertex *v*_*i*_ at *T*_*ij*_ time point. In this study, the active probability set *P* includes three values *P*_*1*_ = 0.99, *P*_*2*_ = 0.95, and *P*_*3*_ = 0.68, respectively. Each PPI *e*(*v*_*i*_,*v*_*j*_) in *E* also has a set of active time attributes *Fe*(*e*(*v*_*i*_, *v*_*j*_)) = *F*_*v*_(*v*_*i*_) ∩ *F*_*v*_(*v*_*j*_) and *Fe*(*e*(*v*_*i*_, *v*_*j*_)) ≠ ∅.

**Fig. 1:**
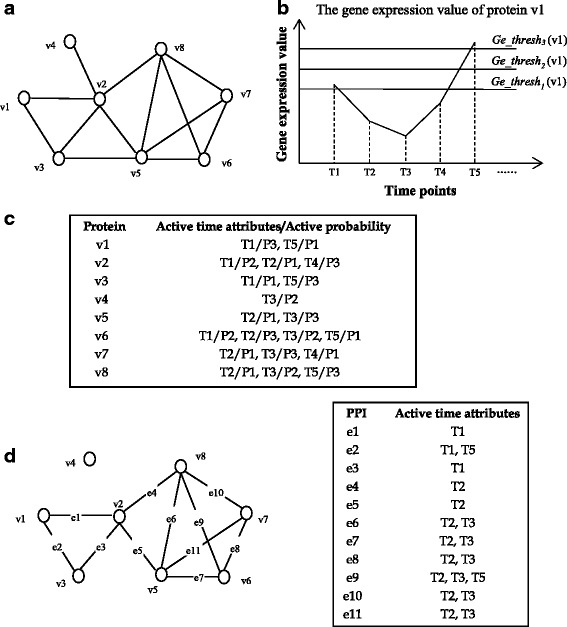
Illustration of DPPN construction. **a** a static PPI network based on high-throughput PPI data. **b** the gene expression value of protein v1. **c** active time attributes and active probability of protein vertices calculated based on gene expression data. **d** a DPPN constructed based on **a** and **c**

Figure 1 shows an example of DPPN construction. Figure 1a is a static PPI networks based on high-throughput PPI data, which consist of eight proteins. Figure 1b shows a part of gene expression value of protein *v*_*1*_. From Fig. 1b, it can be seen that the gene expression value at *T1* and *T5* protein *v*_*1*_ are significantly higher than at *T2*, *T3* and *T4.* According to the equation (4), *Ge_thresh*_*2*_ > G_T1_(*v*_*1*_) > *Ge_thresh*_*1*_ at the time point *T1*, and G_T5_(*v*_*1*_) > *Ge_thresh*_*3*_ at the time point *T5*. Therefore, the active probability of protein *v*_*1*_ are *P3* (0.68) and *P1* (0.99) at the time point *T1* and *T5,* respectively. Figure 1c lists the active time attributes and active probability of all protein vertices in Fig. 1a. It can be seen that each protein vertex has an active time attribute set. For instance, *v*_*1*_ has two active time attributes (*T1* and *T5*), and *v*_*2*_ has three active time attributes (*T1, T2* and *T4*). In particular, each protein vertex has an active probability at an active time attribute. In Fig. 1c, the active probability of *v*_*1*_ is *P3* (0.68) and *P1* (0.99) at the *T1* and *T5* time points, respectively. Figure 1d shows a DPPN constructed based on Fig. 1a and c. Each edge in DPPN has an active time attributes set. For example, *e*_*1*_ represents the PPI between *v*_*1*_ and *v*_*2*_*.* The active time attributes sets of *v*_*1*_ and *v*_*2*_ are {*T1,T5*} and {*T1, T2, T4*} based on Fig. 1c, respectively. The active time attribute set of *e*_*1*_ is {*T1*} which is calculate by {*T1, T5*}∩{*T1, T2, T4*}. If the active time attribute set of an edge is empty, the edge will not appear in DPPN.

### Protein complex identification from DPPN

Compared to static PPI networks, DPPN can effectively represent not only the topological structure but also the dynamic information of PPI networks. Since protein complexes are groups of proteins that interact with each other in the same time [2, 3], they are generally dense subgraph associated with the same active time attributes in DPPN. The edges in DPPN contribute differently for protein complex identification task. Given a DPPN *G*, the topology score of edge *e(v*_*i*_*,v*_*j*_*)* is defined as follows:

5 $$ \mathrm{Topology}\_\mathrm{score}\left(e\left(vi,vj\right)\right)=\frac{\left|{N}_i\cap {N}_j\right|+1}{ \max \left\{Avg.(G),\left|{N}_i\right|\right\}+ \max \left\{Avg.(G),\left|{N}_j\right|\right\}} $$

6 $$ Avg.(G)=\frac{{\displaystyle {\sum}_{v_k\in V}\left|{N}_k\right|}}{\left|V\right|} $$

where *N*_*i*_ and *N*_*j*_ denote the neighbors of *v*_*i*_ and *v*_*j*_ respectively. |*N*_*i*_ ∩*N*_*j*_ | denotes the common neighbors of *v*_*i*_ and *v*_*j*_, and *Avg.(G)* calculates the average degree of the DPPN *G*. If *v*_*i*_ and *v*_*j*_ share more common neighbors, the topology score will be larger. Max{*Avg.(G)*, |*N*_*i*_|}can penalize protein *v*_*i*_ with very few neighbors effectively [10]. Based on the topology weight, the weight of edge *e(v*_*i*_*,v*_*j*_*)* at the *k* active time point is given as:

7 $$ \mathrm{Weight}\left(ek\left(vi,vj\right)\right)=\mathrm{Topology}\_\mathrm{score}\left(e\left(vi,vj\right)\right)\cdotp Pk(vi)\cdotp Pk(vj) $$

where P_*k*_(*v*_*i*_) and P_*k*_(*v*_*j*_) are the active probability of *v*_*i*_ and *v*_*j*_ at the *k* time point, respectively. The equation (7) can consider not only the topological structure but also the dynamic information of DPPN. Since the active probability of *v*_*i*_ and *v*_*j*_ is likely different at different active time point, the weight of edge *e(v*_*i*_*,v*_*j*_*)* dynamically changes during all active time points.

Definition 1 - Active correlated clique. Given a protein vertex set *C* and an edge set *E*_*c*_ in DPPN *G*, an active correlated clique is a pair ((C, *E*_*c*_), *A*_*c*_), such that for each protein vertex *v*_*i*_ in *C*, the degree of *v*_*i*_ is |*C|*-1. *A*_*c*_ is the common active time attribute set of each protein vertex *v*_*i*_ in *C* and *Ac* ≠ ∅.

**Fig. 2:**
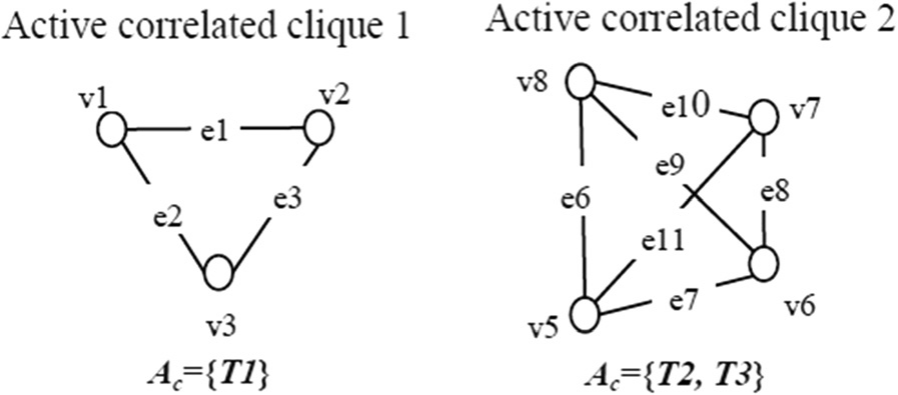
Examples of active correlated cliques

In general, we can mine many Active correlated cliques in a DPPN. Figure 2 shows two active correlated cliques of the DPPN in Fig. 1.

Definition 2 – Active clique score. Given an active correlated clique ((C, *E*_*c*_), *A*_*c*_), the Active clique score of ((C, *E*_*c*_), *A*_*c*_) at the *k* (*k*∈*A*_*c*_) active time point, is given as:

8 $$ \mathrm{Clique}\_\mathrm{score}\left(\left(C,Ec\right),Ac\right)=\mathrm{Clique}\_ \Pr .\left(\left(C,Ec\right),Ac\right)\cdotp {\displaystyle {\sum}_{eij\in Ec}\mathrm{Toplogy}\_\mathrm{score}(eij)} $$

9 $$ \mathrm{Clique}\_ \Pr .\left(\left(C,Ec\right),Ac\right)= \max \left\{{\displaystyle {\prod}_{vi\in C}Pk}(vi),k\in Ac\right\} $$

where P_*k*_(*v*_*i*_) is the active probability of *v*_*i*_ at the *k* time point. ∏_*vi* ∈ *C*_*Pk*(*vi*) calculates the active probability of clique((C, *E*_*c*_), *A*_*c*_) at the *k* time point. Clique_Pr. ((C, *E*_*c*_), *A*_*c*_) choose the maximum ∏_*vi* ∈ *C*_*Pk*(*vi*) as the active probability for the clique from all the common active time points. Therefore, active probability of an active correlated clique is associated with an unique active time point. We can use ((C, *E*_*c*_), *T*_*c*_) to denote an active correlated clique which gets the clique probability at *T*_*c*_ active time point. Clique score provides a reasonable combination of topology connectivity and the dynamic active attributes of DPPN. If an active correlated clique is associated with a large clique score, this indicates that the proteins of the clique are all in dense subgraph structure of DPPN as well as highly active at a same time point. Therefore, the clique score can effectively evaluate how possible an active correlated clique is the core structure of a protein complex.

Gavin et al*.* [11] revealed the core-attachment structure of protein complex by genome-wide analyzing yeast complexes. Based on core-attachment structure assumption, our method for protein complex identification from DPPN involved two phases. In the first phases, we identified the core structure of protein complexes from DPPN. In the second phases, we augmented the protein complex from the core structure by adding the close neighbor proteins.

In the first phase, we used the cliques mining algorithm [27] to enumerate all maximal cliques which contain three or more proteins from DPPN, and calculated the common active time attribute set for each maximal clique. If the common active time attribute set was not empty, the maximal clique was an active correlated clique. The candidate core set *Candidate_CORE* was comprised of all active correlated cliques, which generally overlapped. We used equation (8) to calculate the active clique score for all active correlated cliques in *Candidate_CORE*, and ranked them in descending order of active clique score, denoted as {((C, *E*_*c1*_), *T*_*c1*_), ((C, *E*_*c2*_), *T*_*c2*_),…,((C, *E*_*cn*_), *T*_*cn*_)}. The top ranked clique((C, *E*_*c1*_), *T*_*c1*_) was then deleted from *Candidate_CORE* and inserted into the core set *CORE*. To ensure that the active correlated cliques in *CORE* were non-overlapping, we used the same method [10] to remove or prune overlapping cliques until the candidate core set *Candidate_CORE* was empty. In this way, we could generate core structures for most protein complexes. However, some protein complexes are with low density or only contain two proteins [28, 29]. To solve this problem, we added some edges with high weight score to the core set *CORE*. We used the equation (7) to calculate the weight for the edges which were not contained in all active correlated cliques. If the weight of an edge was larger than the predefined threshhold *core_thresh*, we directly added the edge to core set *CORE*. Therefore, we chose not only active correlated cliques but also the edges associated with high weight score as core structures of protein complexes.

In the second phase, we augmented the core structure by adding each close neighbor protein one by one. We used attached score to measure how closely a protein *v*_*k*_ with active time attribute *A*_*k*_ was connected to a core structure ((C, *E*_*c*_), *T*_*c*_), where *vk* ∉ *C* and *Tc* ∈ *Ak*. The attached score of *v*_*k*_ with respect to ((C, *E*_*c*_), *T*_*c*_) is given as:

10 $$ Attach\_\mathrm{score}\left(\left(vk,Ak\right),\left(\left(C,Ec\right),Tc\right)\right)=\frac{{\displaystyle {\sum}_{vi\in C} Weight\left({e}_{Tc}\left(vi,vk\right)\right)}}{\left|C\right|} $$

If the Attach_score was larger than *extend_thresh*, then *v*_*k*_ was added to the core structure ((C, *E*_*c*_), *T*_*c*_). Therefore the final identified protein complexes were generated by adding the close neighbor proteins to the core structure. Here, *extend_thresh* was a predefined threshold. The optimal value of *extend_thresh* and *core_thresh* can usually be determined in preliminary experiments.

## Results and discussion

In this section, the datasets and evaluation metrics used in the experiments are described. The impact of the *core_thresh and extend_thresh* parameters are assessed. Finally, our method is compared with current state-of-the-art protein complex identification methods.

### Datasets and evaluation metrics

The three high-throughput PPI datasets used in our experiment were the Krogan dataset [30], DIP dataset [31] and MIPS dataset [32], respectively. The statistics of the three yeast PPI datasets is listed in Table 1. The benchmark protein complex datasets are CYC2008 [28] and MIPS2006 [33], which consist of 408 and 217 protein complexes, respectively.

**Table 1:** The statistics of high-throughput PPI datasets in experiments

PPI datasets	Proteins	Interactions
Krogan dataset	2675	7080
DIP dataset	4928	17208
MIPS dataset	3950	11119

**Fig. 3:**
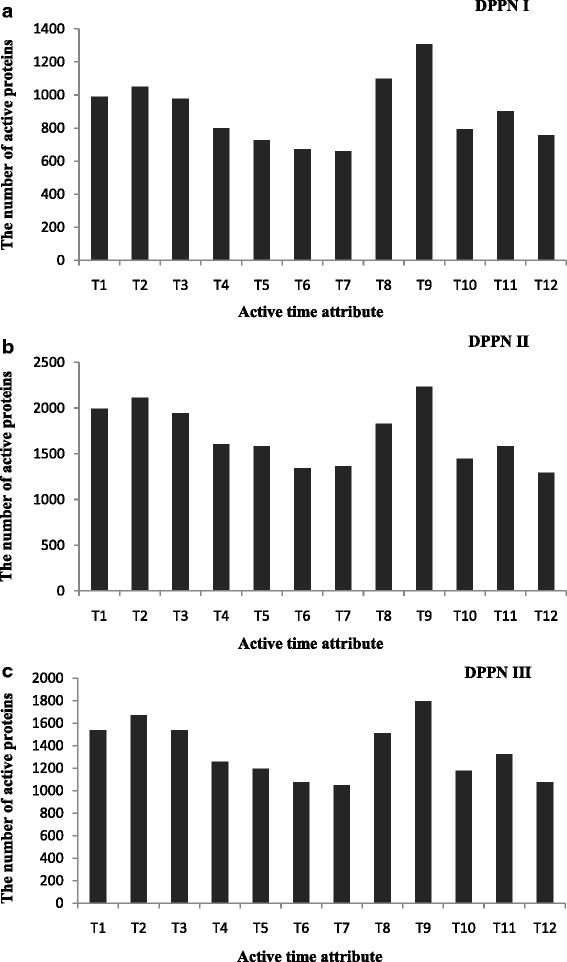
The distribution of the number of active proteins. **a**, **b** and **c** are the distribution of the number of active proteins in DPPN I, DPPN II and DPPN III, respectively

The gene expression data used in our experiment was GSE3431 [34] downloaded from Gene Expression Omnibus (GEO), which is an expression profiling of yeast by array affymetrix gene expression data over three successive metabolic cycles. GSE3431 gene expression data is 12 time intervals per cycle. Therefore, there are 12 active time points (*T1,T2,…,T12*) for each gene in a cycle. We constructed three DPPN networks to integrate high-throughput PPI data and gene expression data as described in the Section “Construction of DPPN”. DPPN I, DPPN II and DPPN III were constructed by integrating gene expression data GSE3431 with the Krogan dataset, DIP dataset and MIPS dataset, respectively. Compared to the static PPI networks, DPPNs could effectively distinguish the active period of a protein by active time attribute of the protein. In this study, if the active probability of a protein higher than or equal to *P3* (0.68) at a time point, the protein is considered as active at that time point. The distributions of the number of active proteins with different active time attributes on DPPN I, DPPN II and DPPN III were given in Fig. 3a, b and c, respectively. We could observe that there was an obvious peak at *T9* in Fig. 3a, b and c. There were 1306, 2234 and 1793 active proteins at *T9* on DPPN I, DPPN II and DPPN III, respectively.

Let *P*(*V*_*P*_*, E*_*P*_) be an identified complex and *B*(*V*_*B*_*, E*_*B*_) be a known complex. We defined the neighborhood affinity score *NA*(*P,B*) between *P*(*V*_*P*_*, E*_*P*_) and *B*(*V*_*B*_*, E*_*B*_) as follows:

11 $$ NA\left(P,B\right)=\frac{{\left|VP\cap VB\right|}^2}{\left|VP\right|\times \left|VB\right|} $$

If *NA*(*P,B*) is 1, it means that the identified complex *P*(*V*_*P*_*, E*_*P*_) has the same proteins as a known complex *B*(*V*_*B*_*, E*_*B*_). On the contrary, if *NA*(*P,B*) is 0, it indicates no shared protein between *P*(*V*_*P*_*, E*_*P*_) and *B*(*V*_*B*_*, E*_*B*_). We considered *P*(*V*_*P*_*, E*_*P*_) and *B*(*V*_*B*_*, E*_*B*_) to match each other if *NA*(*P,B*) was larger than 0.2, which is the same as most methods for protein complex identification [3].

Precision, recall and *F-score* have been used to evaluate the performance of protein complex identification methods by most previous studies. The definitions of precision, recall and *F-score* are given as follows:

12 $$ precision=\frac{Ncp}{\left| Identified\_ Set\right|} $$

13 $$ recall=\frac{Ncb}{\left| Benchmark\_ Set\right|} $$

14 $$ F\hbox{-} score=\frac{2 precision\cdot recall}{\left( precision+ recall\right)} $$

where *N*_*cp*_ is the number of identified complexes which match at least one known complex and *N*_*cb*_ is the number of known complexes that match at least one identified complex. *Identified_Set* denotes the set of complexes identified by a method and *Benchmark_Set* denotes the reference benchmark set. Precision measures the fidelity of the identified protein complex set. Recall quantifies the extent to which a identified complex set captures the known complexes in the benchmark set. *F-score* provides a reasonable combination of both precision and recall, and can be used to evaluate the overall performance.

Recently, sensitivity (Sn), positive predictive value (PPV) and accuracy (Acc) have also been used to evaluate protein complex identification tools. Given *n* benchmark complexes and *m* identified complexes, let *T*_*ij*_ denote the number of proteins in common between *i*_*th*_ benchmark complex and *j*_*th*_ identified complex. Sn and PPV are then defined as follows:

15 $$ Sn=\frac{{\displaystyle {\sum}_{i=1}^n{ \max}_{j=i}^m\left\{{T}_{ij}\right\}}}{{\displaystyle {\sum}_{i=1}^n{N}_i}} $$

16 $$ PPV=\frac{{\displaystyle {\sum}_{j=1}^m{ \max}_{i=1}^n}\left\{{T}_{ij}\right\}}{{\displaystyle {\sum}_{j=1}^m{\displaystyle {\sum}_{i=j}^n{T}_{ij}}}} $$

Here *N*_*i*_ is the number of proteins in the *i*_*th*_ benchmark complex. Generally, high Sn value indicates that the prediction has a good coverage of the proteins in the benchmark complexes, while high PPV value indicates that the predicted complexes are likely to be true positives. Accuracy is the geometrical mean of the Sn and PPV, which is defined as follows:

17 $$ Accuracy=\sqrt{Sn\kern0.5em PPV} $$

Accuracy represents a tradeoff between Sn and PPV value. The advantage of taking the geometric mean is that it yields a low score when either the Sn or PPV metric is low. High accuracy values thus require a high performance for both criteria. To keep in line with most previous studies, we chose precision, recall and *F-score* as the major evaluate measures in this study, and also reported Sn, PPV and Accuracy.

### The effect of threshhold parameters

In this experiment, we evaluated the effect of the threshold parameters of our method on the DPPN I. The parameters, *extend_thresh* and *core_thresh*, range from 0 to 1. We can choose the optimal value of *extend_thresh* and *core_thresh* by the experimental approach. Firstly, we kept *extend_thresh* =0.05 and evaluated the effect of *core_thresh*. The detailed experimental results on the DPPN I with different *core_thresh* were shown in Table 2. The highest value in each row was shown in bold.

**Table 2:** The effect of “*Core_thresh*” on DPPN I

*Core_thresh*	P	R	F	Sn	PPV	Acc
0	0.357	**0.574**	0.44	**0.395**	0.75	0.544
0.02	0.357	**0.574**	0.44	**0.395**	**0.751**	**0.545**
0.04	0.364	0.564	0.443	0.393	0.748	0.542
0.06	0.38	0.547	0.448	0.388	0.746	0.538
0.07	0.41	0.517	0.458	0.378	0.741	0.529
0.08	0.424	0.5	0.459	0.374	0.735	0.524
0.09	0.468	0.475	**0.471**	0.364	0.729	0.515
0.1	0.562	0.338	0.422	0.307	0.713	0.468
0.2	0.621	0.301	0.406	0.306	0.706	0.464
0.5	**0.718**	0.297	0.42	0.313	0.702	0.469
1.0	**0.718**	0.297	0.42	0.313	0.702	0.469

F: F-score, P: precision, R: recall. The highest score of each row is shown in bold

As shown in Table 2, when *core_thresh* was too small, many edges with low weight score would be added to core set. This would lead to identify many false protein complexes and degrade the *F-score* of our method. On the contrary, when *core_thresh* was too large, little edges would be added to core set even though some edges with high weight score. Overall, our method achieved the highest *F-score*, when *core_thresh* =0.09.

Secondly, we kept *core_thresh* =0.09 and evaluated the effect of *extend_thresh.* The detailed experimental results on DPPN I with different *extend_thresh* were shown in Table 3. The highest value in each row was shown in bold. It can be seen that our method proved sensitive to *extend_thresh* between 0 and 0.1. *F-score* performance ranged from 0.433 to 0.471. When *extend_thresh* = 0, precision, recall and *F-score* were 0.428, 0.439 and 0.433, respectively. As *extend_thresh* was increased, the number of proteins added decreased sharply. When *extend_thresh* = 0.05, precision, recall and *F-score* achieved 0.468, 0.475 and 0.471, respectively. When *extend_thresh* was increased from 0.05 to 0.1, precision, recall and *F-score* all decreased.

**Table 3:** The effect of “*Extend_thresh*” on DPPN I

*Extend_thresh*	P	R	F	Sn	PPV	Acc
0	0.428	0.439	0.433	**0.421**	0.629	0.515
0.02	0.463	**0.475**	0.469	0.39	0.702	**0.523**
0.04	**0.468**	0.468	0.468	0.372	0.724	0.519
0.05	**0.468**	**0.475**	**0.471**	0.364	0.729	0.515
0.06	0.465	**0.475**	0.47	0.36	0.734	0.514
0.08	0.46	0.471	0.465	0.351	0.739	0.509
0.1	0.458	0.468	0.463	0.346	0.741	0.507
0.2	0.455	0.468	0.461	0.336	**0.744**	0.5
0.5	0.45	0.468	0.459	0.334	0.743	0.498
1.0	0.45	0.468	0.459	0.334	0.743	0.498
0	0.428	0.439	0.433	**0.421**	0.629	0.515

F: F-score, P: precision, R: recall. The highest score of each row is shown in bold

Then we evaluated Sn, PPV and Acc metrics for *extend_thresh* on DPPN I in Table 3. When *extend_thresh* was changed from 0 to 0.1, PPV increased whereas Sn decreased. When *extend_thresh* ranged between 0.1 and 1.0, Sn, PPV and Acc did not change appreciably. Acc was defined as the geometric mean of Sn and PPV, which was maximized (0.523) when *extend_thres* = 0.02. Compared with Acc, *F-score* is more effectively and reasonably to evaluate the performance of a method. From Table3, it can be seen that our method can achieve highest *F-score*, when *extend_thresh* =0.05.

### Comparison with other methods

We compared our method on three DPPNs with the following state-of-the-art protein complex identification methods (Tables 4, 5, 6, 7, 8 and 9): ClusterONE [15], COAN [16], COACH [12], CMC [10], HUNTER [14] and MCL [5]. To equally compare the performance, we test all comparison methods on the Krogan, DIP and MIPS dataset respectively, and use the CYC2008 as benchmark dataset to choose the optimal parameters. For our method, the parameter *core_thresh* and *extend_thresh* is set to 0.09 and 0.05, respectively. For ClusterONE, the “*Overlap”* parameter is set to 0.8. For COAN, the *“Threshold”* parameter is set to 0.6. For COACH, the *“Omega”* parameter is set to 0.2. For CMC, the *“overlap_thres”* and *“merge_thres”* parameters are set to 0.5 and 0.25, respectively. For MCL, the “*inflation*” parameter is set to 2.5. The highest value in each row was shown in bold.

**Table 4:** Performance comparison with other methods on Krogan dataset using CYC2008 as benchmark

	P	R	F	Sn	PPV	Acc
Our method	0.468	**0.475**	**0.471**	0.364	**0.729**	0.515
ClusterONE	0.375	0.431	0.401	0.523	0.655	**0.585**
COAN	0.709	0.331	0.451	0.388	0.646	0.501
COACH	0.617	0.343	0.441	0.432	0.544	0.485
CMC	0.748	0.235	0.358	0.381	0.589	0.474
HUNTER	**0.865**	0.199	0.323	0.374	0.569	0.462
MCL	0.291	0.245	0.266	**0.57**	0.396	0.475

F: F-score, P: precision, R: recall. The highest score of each row is shown in bold

**Table 5:** Performance comparison with other methods on DIP dataset using CYC2008 as benchmark

	P	R	F	Sn	PPV	Acc
Our method	0.483	**0.471**	**0.477**	0.373	**0.694**	**0.509**
ClusterONE	0.428	0.331	0.373	0.364	0.665	0.493
COAN	0.486	0.438	0.461	0.435	0.555	0.491
COACH	0.364	0.468	0.41	0.544	0.38	0.455
CMC	0.595	0.287	0.387	0.399	0.566	0.475
HUNTER	**0.685**	0.199	0.308	0.496	0.467	0.482
MCL	0.21	0.232	0.221	**0.555**	0.331	0.429

F: F-score, P: precision, R: recall. The highest score of each row is shown in bold

**Table 6:** Performance comparison with other methods on MIPS dataset using CYC2008 as benchmark

	P	R	F	Sn	PPV	Acc
Our method	0.467	**0.324**	**0.382**	0.245	0.662	**0.403**
ClusterONE	0.359	0.23	0.281	0.243	**0.668**	**0.403**
COAN	0.453	0.282	0.348	0.271	0.55	0.386
COACH	0.301	0.289	0.295	0.336	0.311	0.323
CMC	0.429	0.211	0.283	0.389	0.318	0.352
HUNTER	**0.654**	0.11	0.189	0.296	0.286	0.291
MCL	0.164	0.154	0.159	**0.444**	0.212	0.307

F: F-score, P: precision, R: recall. The highest score of each row is shown in bold

**Table 7:** Performance comparison with other methods on Krogan dataset using MIPS2006 as benchmark

	P	R	F	Sn	PPV	Acc
Our method	0.22	**0.424**	0.285	0.293	**0.726**	0.461
ClusterONE	0.317	0.327	0.322	0.328	0.667	0.467
COAN	0.46	0.35	**0.398**	0.352	0.696	**0.495**
COACH	0.357	0.341	0.349	0.357	0.673	0.49
CMC	0.309	0.304	0.306	0.401	0.569	0.478
HUNTER	**0.473**	0.207	0.288	0.317	0.602	0.437
MCL	0.149	0.23	0.181	**0.485**	0.444	0.464

F: F-score, P: precision, R: recall. The highest score of each row is shown in bold

**Table 8:** Performance comparison with other methods on DIP dataset using MIPS2006 as benchmark

	P	R	F	Sn	PPV	Acc
Our method	0.292	0.535	0.378	0.325	**0.718**	0.483
ClusterONE	0.246	0.392	0.302	0.321	0.623	0.447
COAN	0.326	0.548	**0.409**	0.397	0.642	**0.505**
COACH	0.289	0.488	0.363	0.452	0.506	0.478
CMC	0.172	**0.58**	0.265	0.367	0.656	0.49
HUNTER	**0.63**	0.097	0.168	0.147	0.555	0.286
MCL	0.121	0.217	0.155	**0.531**	0.382	0.451

F: F-score, P: precision, R: recall. The highest score of each row is shown in bold

**Table 9:** Performance comparison with other methods on MIPS dataset using MIPS2006 as benchmark

	P	R	F	Sn	PPV	Acc
Our method	0.336	**0.401**	**0.372**	0.24	0.683	0.405
ClusterONE	0.281	0.327	0.302	0.262	**0.69**	**0.426**
COAN	0.343	0.366	0.358	0.303	0.515	0.395
COACH	0.286	0.373	0.286	0.333	0.359	0.346
CMC	0.299	0.318	0.308	0.381	0.473	0.424
HUNTER	**0.462**	0.138	0.213	0.298	0.341	0.319
MCL	0.108	0.194	0.139	**0.451**	0.266	0.347

F: F-score, P: precision, R: recall. The highest score of each row is shown in bold

Tables 4, 5 and 6 listed the performance comparison results using CYC2008 as benchmark. Firstly, we compared our method using DPPN I with ClusterONE, COACH, CMC, HUNTER and MCL using the Krogan PPI network. As shown in Table 4, ClusterONE achieved the highest Acc of 0.585. HUNTER and MCL achieved the highest precision of 0.865 and the highest Sn of 0.57, respectively. Compared with other methods, our method achieved the highest *F-score* of 0.471, the highest recall of 0.475 and the highest PPV of 0.729, which was significantly superior to the other methods. Secondly, we compared our method using DPPN II with the other methods using the DIP PPI network. From Table 5, it could be seen that our method achieved both the highest *F-score* of 0.477 and the highest Acc of 0.509. HUNTER and MCL also achieved the highest precision (0.685) and the highest Sn (0.555) in Table 5. Thirdly, we compared our method using DPPN III with the other methods using the MIPS PPI network. Similarly, our method also achieved both the highest *F-score* of 0.382 and the highest Acc of 0.403 in Table 6.

Table 7, 8 and 9 listed the performance comparison results using MIPS2006 as the benchmark. From Table 7, our method achieved the highest recall of 0.424 and the highest PPV of 0.726, respectively. COAN achieved the highest *F-score* of 0.398 and the highest Acc of 0.495, respectively. From Table 8, our method also achieved the highest PPV of 0.718 and a high *F-score* of 0.378. COAN also achieved the highest *F-score* of 0.409 and the highest Acc of 0.505, respectively. Form Table 9, our method achieved the highest recall of 0.401 and the highest *F-score* of 0.372, respectively. ClusterONE achieved the highest Acc of 0.426 in the Table 9. We also noted that the performance results of most comparison methods using MIPS2006 as benchmark were inferior to the performance results using CYC2008 as benchmark in Tables 7 and 8. For instance, our method achieved a low *F-score* of 0.285 in the Table 7, which was significantly inferior to the *F-score* of 0.471 in the Table 4. The main reason was that the comparison methods used CYC2008 as benchmark to choose the optimal parameters.

Next, we compared our method with DyCluster [25] in the Tables 10, 11, 12, 13, 14 and 15. DyCluster is a framework to detect complexes based on PPI data and gene expression data, which was proposed by Hanna et al*.* DyCluster uses biclustering techniques to construct dynamic PPI networks by incorporating gene expression data, and then applies the existing complex-detection algorithms, such as ClusterONE and CMC, to detect the complexes from the dynamic PPI networks. Based on DyCluster framework, we can compare our method with existing methods that integrate gene expression data with PPI data. In the Tables 10, 11, 12, 13, 14 and 15, “DyCluster + ClusterONE” denotes using DyCluster framework to construct dynamic PPI networks and applying ClusterONE method to predict complexes from dynamic PPI networks. In Tables 10, 11 and 12, we used CYC2008 as the benchmark. It can be seen that our method achieved the highest *F-score* in Tables 10, 11 and 12, and “DyCluster + ClusterONE” achieved the highest Acc in Tables 10 and 12. In Tables 13, 14 and 15, we used MIPS2006 as the benchmark. Similarly, our method and “DyCluster + ClusterONE” achieved the highest *F-score* and Acc in Tables 13, 14 and 15, respectively.

**Table 10:** Performance comparison with DyCluster method on Krogan dataset and gene expression data using CYC2008 as benchmark

	P	R	F	Sn	PPV	Acc
Our method	0.468	**0.475**	**0.471**	0.364	**0.729**	0.515
DyCluster + ClusterONE	0.307	0.348	0.326	**0.394**	0.682	**0.518**
DyCluster + COAN	0.565	0.23	0.327	0.27	0.677	0.428
DyCluster + COACH	0.48	0.243	0.322	0.321	0.617	0.445
DyCluster + CMC	0.531	0.201	0.292	0.258	0.691	0.423
DyCluster + HUNTER	**0.569**	0.169	0.261	0.268	0.493	0.364
DyCluster + MCL	0.29	0.173	0.214	0.371	0.376	0.373

F: F-score, P: precision, R: recall. The highest score of each row is shown in bold

**Table 11:** Performance comparison with DyCluster method on DIP dataset and gene expression data using CYC2008 as benchmark

	P	R	F	Sn	PPV	Acc
Our method	**0.483**	**0.471**	**0.477**	0.373	**0.694**	**0.509**
DyCluster + ClusterONE	0.153	0.373	0.217	0.399	0.63	0.501
DyCluster + COAN	0.349	0.311	0.329	0.339	0.596	0.449
DyCluster + COACH	0.319	0.375	0.344	0.409	0.54	0.47
DyCluster + CMC	0.316	0.294	0.305	0.328	0.565	0.43
DyCluster + HUNTER	0.472	0.147	0.224	0.226	0.618	0.374
DyCluster + MCL	0.243	0.228	0.237	**0.497**	0.343	0.413

F: F-score, P: precision, R: recall. The highest score of each row is shown in bold

**Table 12:** Performance comparison with DyCluster method on MIPS dataset and gene expression data using CYC2008 as benchmark

	P	R	F	Sn	PPV	Acc
Our method	**0.467**	**0.324**	**0.382**	0.245	**0.662**	0.403
DyCluster + ClusterONE	0.157	0.27	0.198	**0.301**	0.597	**0.424**
DyCluster + COAN	0.39	0.216	0.278	0.223	0.601	0.366
DyCluster + COACH	0.304	0.216	0.252	0.24	0.522	0.354
DyCluster + CMC	0.363	0.174	0.235	0.199	0.572	0.337
DyCluster + HUNTER	0.421	0.123	0.19	0.195	0.527	0.321
DyCluster + MCL	0.156	0.11	0.129	0.232	0.275	0.253

F: F-score, P: precision, R: recall. The highest score of each row is shown in bold

**Table 13:** Performance comparison with DyCluster method on Krogan dataset and gene expression data using MIPS2006 as benchmark

	P	R	F	Sn	PPV	Acc
Our method	0.22	**0.424**	**0.285**	0.293	0.726	0.461
DyCluster + ClusterONE	0.149	0.341	0.208	0.332	**0.736**	**0.494**
DyCluster + COAN	**0.305**	0.244	0.271	0.249	0.699	0.417
DyCluster + COACH	0.267	0.272	0.27	0.285	0.663	0.435
DyCluster + CMC	0.269	0.212	0.237	0.221	0.706	0.395
DyCluster + HUNTER	0.294	0.161	0.208	0.218	0.501	0.331
DyCluster + MCL	0.149	0.23	0.181	**0.467**	0.43	0.448

F: F-score, P: precision, R: recall. The highest score of each row is shown in bold

**Table 14:** Performance comparison with Dycluster method on DIP dataset and gene expression data using MIPS2006 as benchmark

	P	R	F	Sn	PPV	Acc
Our method	0.292	**0.535**	**0.378**	0.325	**0.718**	0.483
DyCluster + ClusterONE	0.094	0.424	0.154	0.358	0.683	**0.494**
DyCluster + COAN	0.245	0.406	0.306	0.317	0.669	0.461
DyCluster + COACH	0.206	0.461	0.284	0.373	0.624	0.483
DyCluster + CMC	0.214	0.369	0.271	0.298	0.631	0.434
DyCluster + HUNTER	**0.324**	0.184	0.235	0.207	0.664	0.371
DyCluster + MCL	0.147	0.207	0.172	**0.443**	0.412	0.428

F: F-score, P: precision, R: recall. The highest score of each row is shown in bold

**Table 15:** Performance comparison with DyCluster method on MIPS dataset and gene expression data using MIPS2006 as benchmark

	P	R	F	Sn	PPV	Acc
Our method	**0.336**	**0.401**	**0.372**	0.24	**0.683**	0.405
DyCluster + ClusterONE	0.118	0.369	0.178	**0.302**	0.659	**0.446**
DyCluster + COAN	0.264	0.276	0.27	0.234	0.611	0.378
DyCluster + COACH	0.196	0.267	0.226	0.247	0.586	0.38
DyCluster + CMC	0.235	0.23	0.233	0.213	0.602	0.358
DyCluster + HUNTER	0.29	0.171	0.215	0.197	0.554	0.33
DyCluster + MCL	0.102	0.143	0.119	0.229	0.304	0.264

F: F-score, P: precision, R: recall. The highest score of each row is shown in bold

Finally, we shuffled the gene expression data and tested whether or not the temporal information in the gene expression data can help identify protein complexes. There are expression levels at 12 time points for each gene in the GSE3431 gene expression data. In this experiment, we took these expression levels for each gene, and shuffled them between the different time points. Each gene retained the same set of gene expression levels, but the order in which these expression level changes happen was now shuffled. We compared the performance of our method on GSE3431 gene expression data with the gene expression data shuffled randomly in the Tables 16 and 17. Form the Tables 16 and 17, it can be seen that the performance of our method on the gene expression data shuffled randomly was significantly inferior to the GSE3431 gene expression data. This indicated that the temporal information in the gene expression data was important to identify complexes.

**Table 16:** Performance comparison of our method on gene expression data shuffled randomly using CYC2008 as benchmark

PPI data	Gene expression data	P	R	F	Sn	PPV	Acc
Krogan	GSE3431	0.468	0.475	0.471	0.364	0.729	0.515
	Shuffled randomly	0.435	0.406	0.421	0.28	0.735	0.453
DIP	GSE3431	0.483	0.471	0.477	0.373	0.694	0.509
	Shuffled randomly	0.438	0.417	0.427	0.301	0.672	0.449
MIPS	GSE3431	0.467	0.324	0.382	0.245	0.662	0.403
	Shuffled randomly	0.427	0.294	0.348	0.201	0.671	0.367

F: F-score, P: precision, R: recall

**Table 17:** Performance comparison of our method on gene expression data shuffled randomly using MIPS2006 as benchmark

PPI data	Gene expression data	P	R	F	Sn	PPV	Acc
Krogan	GSE3431	0.22	0.424	0.285	0.293	0.726	0.461
	Shuffled randomly	0.207	0.355	0.262	0.212	0.73	0.393
DIP	GSE3431	0.292	0.535	0.378	0.325	0.718	0.483
	Shuffled randomly	0.253	0.461	0.326	0.253	0.693	0.419
MIPS	GSE3431	0.336	0.401	0.372	0.24	0.683	0.405
	Shuffled randomly	0.273	0.355	0.309	0.197	0.689	0.369

F: F-score, P: precision, R: recall

In summary, our approach achieved the state-of-the-art performance on three DPPNs, which was competitive or superior to the existing protein complexes identification methods. Compared with the prior works, DPPN can not only effectively identify the active time point of the protein, but also distinguish the active level of the protein. The experimental results indicated that DPPN could effectively integrate dynamic information of protein into static PPI networks, and improve the performance of protein complex identification.

### Golgi transport complex identified by our method

**Fig. 4:**
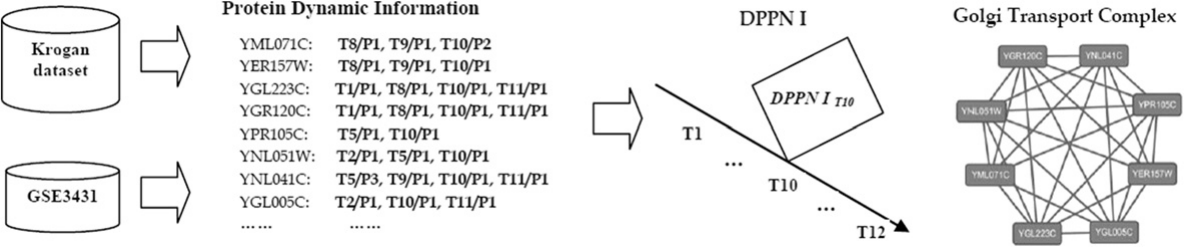
Golgi Transport Complex identified on DPPN I

Figure 4 shows the Golgi Transport Complex identified by our method on DPPN I. Golgi Transport Complex was first found by Whyte et al. through experimental method [35]. They firstly identified the key protein, YML071C, that was involved in vesicle targeting to the yeast Golgi apparatus, and then found it to be associated with seven other proteins. Eventually, Whyte et al. found the Golgi Transport Complex was comprised of eight proteins including YML071C, YER157W, YGL223C, YGR120C, YPR105C, YNL051W, YNL041C and YGL005C.

From Fig. 4, our method firstly calculated the protein dynamic information and constructs the DPPN I based on the gene expression data and MIPS dataset. It can be seen that the eight proteins share the common active time point *T10*. This indicates that all these proteins will be active on the DPPN I at the active time point *T10*. Eventually, our method not only considered the topology information of high-throughput PPI dataset but also the dynamic information of gene expression data to identify the Golgi Transport Complex exactly from DPPN I. Furthermore, this result suggested that the life period of the Golgi Transport Complex is mostly at *T10* time point. Compared with other methods, our method can integrate the dynamic information of gene expression data to improve the performance of protein complex identification, and distinguish the active time point of the identified protein complexes during the cell cycle.

## Conclusions

A challenging task in post-genomic era is to construct dynamic PPI networks and identify protein complex from dynamic PPI networks. In this paper, we first proposed active probability-based method to distinguish the active level of proteins. Based on this, we constructed DPPN to integrate the dynamic information of gene expression data into static PPI networks. Compared with static PPI networks, DPPN could effectively represent the dynamic information as well as topological structure of proteins. Furthermore, we developed a novel method to identify protein complex on DPPN. Experimental comparisons on three DPPNs showed that this approach outperformed established leading protein complex identification tools. The model and the construction method of DPPN could not only be applied to identify protein complex, but also provide a framework to integrate dynamic information into static networks for other applications, such as pathway analysis.

Using gene expression data to construction dynamic PPI networks is based on the assumption that gene expression and protein expression are well correlated. Some studies have suggested that protein levels are not proportional to mRNA levels [36], which can be amplified by post-transcriptional processes. In the future work, we will study how to construct dynamic PPI networks more accurately. We will choose several complexes prediction methods which complement each other, and attempt to combine these methods to predict complexes. In addition, other data and models could further improve complexes prediction. For example, protein location data can be further incorporated into the dynamic PPI networks, which could benefit the complexes identification. We will also try using uncertain graph model to identify the complexes on the dynamic PPI networks.
